# Combinatorial Contextualization of Peptidic Epitopes for Enhanced Cellular Immunity

**DOI:** 10.1371/journal.pone.0110425

**Published:** 2014-10-24

**Authors:** Masaki Ito, Kazumi Hayashi, Eru Adachi, Tamiko Minamisawa, Sadamu Homma, Shigeo Koido, Kiyotaka Shiba

**Affiliations:** 1 Department of Oncology, The Jikei University School of Medicine, Tokyo, Japan; 2 Division of Protein Engineering, Cancer Institute, Japanese Foundation for Cancer Research, Tokyo, Japan; 3 Division of Gastroenterology and Hepatology, Department of Internal Medicine, The Jikei University School of Medicine, Tokyo, Japan; The University of Texas Medical School at Houston, United States of America

## Abstract

Invocation of cellular immunity by epitopic peptides remains largely dependent on empirically developed protocols, such as interfusion of aluminum salts or emulsification using terpenoids and surfactants. To explore novel vaccine formulation, epitopic peptide motifs were co-programmed with structural motifs to produce artificial antigens using our “motif-programming” approach. As a proof of concept, we used an ovalbumin (OVA) system and prepared an artificial protein library by combinatorially polymerizing MHC class I and II sequences from OVA along with a sequence that tends to form secondary structures. The purified endotoxin-free proteins were then examined for their ability to activate OVA-specific T-cell hybridoma cells after being processed within dendritic cells. One clone, F37A (containing three MHC I and two MHC II OVA epitopes), possessed a greater ability to evoke cellular immunity than the native OVA or the other artificial antigens. The sensitivity profiles of drugs that interfered with the F37A uptake differed from those of the other artificial proteins and OVA, suggesting that alteration of the cross-presentation pathway is responsible for the enhanced immunogenicity. Moreover, F37A, but not an epitopic peptide, invoked cellular immunity when injected together with monophosphoryl lipid A (MPL), and retarded tumor growth in mice. Thus, an artificially synthesized protein antigen induced cellular immunity in vivo in the absence of incomplete Freund's adjuvant or aluminum salts. The method described here could be potentially used for developing vaccines for such intractable ailments as AIDS, malaria and cancer, ailments in which cellular immunity likely play a crucial role in prevention and treatment.

## Introduction

Adjuvants are agents that enhance immune responses when co-administered with antigens. Despite their indispensability, the molecular mechanism by which adjuvants boost immune responses is not fully understood [1]. In animal experiments, the most effective adjuvant is complete Freund's adjuvant (CFA) [2], which is composed of inactivated *Mycobacteria* in an oil emulsion often termed “incomplete Freund's adjuvant” (IFA). It is thought the efficacy of CFA may stem from the ability of certain mycobacterial components to activate pattern recognition receptor, such as the Toll-like receptor, and to maturate antigen presenting cells (APCs); while IFA, a mixture of non-metabolizable mineral oil and a surfactant, is thought to accelerate antigen uptake by APCs. Thus from a functional viewpoint, CFA is composed of two modules: a “signal adjuvant” and a “physical adjuvant.”

While CFA has proven to be a superior adjuvant in preclinical experiments, its usage in humans has not been approved due to the likelihood of serious adverse events. As an alternative, aluminum salt has been approved for use in various vaccines, but it is a poor inducer of Th1 cellular responses [3]. MF59 and AS03 are the other approved physical adjuvants. They are composed of an oil emulsion and have been used in some vaccines [4]. The approval of IFA (Montanide ISA 51VG) has been delayed due to the poor definition of its ingredients [5], [6]. As a signal adjuvant, monophosphoryl lipid A (MPL), a derivative of lipopolysaccharide (LPS), is approved for use in vaccines in combination with aluminum salt [7]. Although physical adjuvants are indispensable for efficient immune induction, less understanding of their mechanism of action and quality control difficulties have hindered their development.

Our aim was to use evolutionary engineering to endow antigenic peptides with the properties of a physical adjuvant. MolCraft is a method for synthesizing multifunctional proteins through combinatorial polymerization of multiple motifs embedded in a single microgene [8]–[11]. Because the proteins created by MolCraft can possess a wide variety of physical properties, we thought that by combining epitope motifs with physical motifs, we would be able to obtain clones with the properties of a physical adjuvant.

In this report, we propose a new strategy for synthesizing physical adjuvant-free vaccines in which peptidic epitopes are combinatorially polymerized along with peptidic structural motifs, after which clones that could elicit cellular immunity with only MPL (signal adjuvant) are selected. Our findings indicate that, by affecting the way APCs process the antigen, certain configurations of the epitope motifs combined with the structural motifs within a polypeptide chain could provide the desired properties of a physical adjuvant to the antigen itself.

## Results and Discussion

### Combinatorial synthesis of artificial proteins

As an immunotherapeutic treatment for cancer, vaccination using a synthetic peptide whose sequence corresponds to an epitope of a tumor antigen has been extensively explored. The conceptual basis of these treatments is that the administered peptides would bind to MHC class I molecules on the surface of APCs and be presented to CTLs. However, Toes *et al.* [12] reported that short peptides also have the potential to bind to MHC molecules of non-professional APCs, which often induces tolerance or anergy in patients. The fact that short peptide-based immunotherapies showed limited therapeutic effect in the majority of clinical studies conducted so far [13], also suggests that simple administration of peptidic epitopes to body fluids may not be sufficient to induce effective cellular immunity.

**Figure 1:**
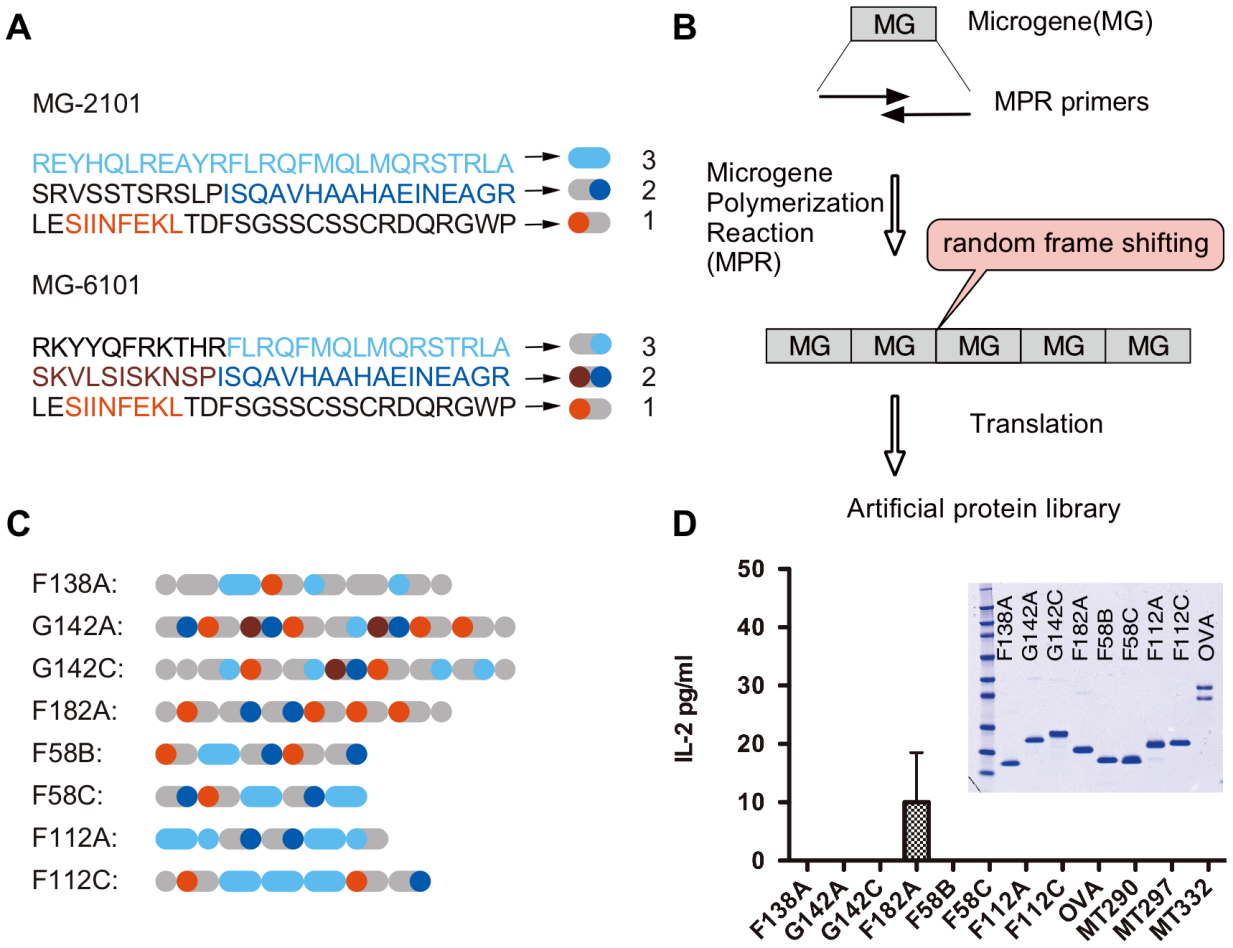
Construction of artificial proteins and *in vitro* antigen presentation assay. (**A**) Amino acid sequences encoded by the microgenes MG2101 and MG6101. Three different reading frames of each microgene encoded three peptides with different amino acid sequences (MG-2101 and MG6101 and Figure S1B, Supporting Information). MHC class I epitope (OVA-I) and class II epitope (OVA-II) are shown in red and blue, respectively. Sequences forming α-helical and β-sheet structures are shown in sky blue and brown, respectively. (**B**) Scheme for generating artificial proteins. Designer microgenes (MG-2101 and MG6101) were polymerized using the microgene polymerization reaction (MPR) [8] with primers designed from the microgene sequences (Figure S1B). These primers contain complementary bases in their 3′ regions and a mismatched base at their 3′-OH ends, which enables efficient polymerization of the microgene. Thermal cycling reactions with MPR primers, the four dNTPs and thermostable exo+ DNA polymerase yields head-to-tail polymers of the microgenes. One intriguing feature of MPR is that the reaction randomly inserts or deletes nucleotides at end-joining junctions, thereby changing the reading frame of the polymers [8]. (**C**) Schematic drawing of the eight artificial proteins used in the initial screening; the amino acid sequences of these proteins are shown in Figure S2, Supporting Information. (**D**) Comparison of the abilities of artificial antigens and native OVA to induce antigen presentation *in vitro*. OVA-specific T-cells were cultured with dendritic cells in the presence of an antigen. The level of IL-2 in the culture supernatant was determined by ELISA. Data shown are mean IL-2 concentration ± standard deviations (SD) (n = 3). Only F182A showed a positive reaction. MT290, MT297 and MT332 are artificial proteins without either OVA-I or OVA-II. Inset: SDS-PAGE of purified artificial proteins.

To overcome these limitations, we employed a method developed by us, known as MolCraft approach, in which epitopic peptide motifs are co-programed with structural motifs to produce an artificial protein library [8]–[11]. For this study, we prepared an artificial protein library by combinatorially combining two epitope motifs from ovalbumin, OVA-I (class I epitope, SIINFEKL) and OVA-II (class II epitope, ISQAVHAAHAEINEAGR), with peptide motifs predicted to form secondary structures (Figure S1A, Supporting Information). The latter motifs were encrypted in the second or third reading frames of the epitope motifs by selecting codons using the program “CyberGene” [10]. One of the resultant microgenes MG-2101 was embedded with a propensity to form α-helix, whereas the other microgene MG-6101 tended to form β-sheet (Figure 1A and Figure S1B, Supporting Information). After generating the artificial protein library using the microgene polymerization reaction (MPR) (Figure 1B), we arbitrarily selected 134 clones, of which 40 (∼28%) were stably expressed in *Escherichia coli*. Although several attempts have been made previously to use recombinant polypeptides composed of simple repeats of epitope sequences [14]–[16], those repetitious proteins were generally unstable and often could not be produced in *E. coli*. Our motif-programming technology solved the problem of stability by co-programming epitopic peptide motifs with structural motifs, which makes it possible to produce stable artificial proteins containing repeating peptidic motifs.

### Selection of artificial proteins that were cross-presented on APCs and that stimulated OVA-specific CTLs *in vitro*

**Figure 2:**
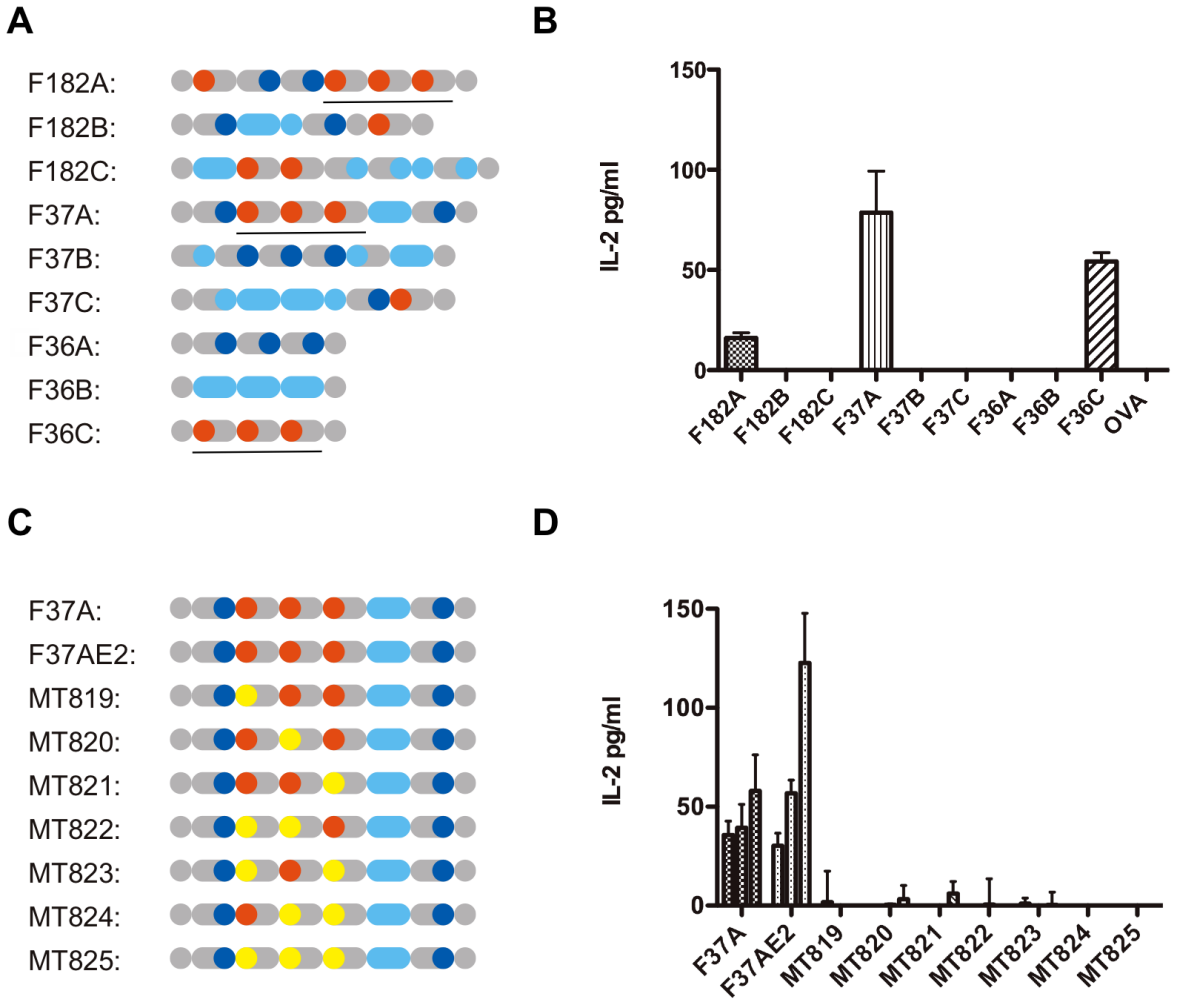
Triple tandem repeats of the sequence LESIINFEKLTDFSGSSCSSCRDQRGWP, which includes the MHC class I epitope (OVA-I), are important for antigen presentation *in vitro*. (**A**) Schematic drawing of nine artificial proteins used in the second screening; the amino acid sequences of these proteins are shown in Figure S3, Supporting Information. Underlines show the common structure (triple tandem repeats of the class I epitope and peptide sequence produced by the cybergene program) present in F182A, F37A and F36C. (**B**) OVA-specific T-cell activation *in vitro* by F37A and F36C. (**C**) Schematic drawings of sequences present in F37A and its mutants. The OVA-I sequences (SIINFEKL) (red) of F37A were replaced with one or more WT1 epitopes (RMFNAPYL) (yellow), one-by-one. (**D**) Determination of OVA-specific antigenicity of the mutant proteins using *in vitro* antigen presentation assay. When DC2.4 cells were treated with different concentrations (10 µg/ml, 20 µg/ml and 40 µg/ml), of the indicated antigens, only F37A and F37AE2, but not the other mutants, induced IL-2 production. Only the five C-terminal amino acids of F37AE2 differed from F37A.

Dendritic cells are known to be professional antigen presenting cells (APCs), which take up exogenous proteins via endocytosis, process them into peptide fragments, and display the cleaved peptides to T-cells by loading them onto major histocompatibility complex (MHC) molecules on the cell surface [17]. Historically, the processing pathway that stimulates humoral immunity has been studied in the context of exogenous antigens, which are endocytosed and processed within the endosomes before being loaded onto MHC class II molecules. Recent studies also revealed that some endocytosed peptides escape from the vesicles into the cytosolic space, where they are further cleaved into smaller pieces by proteasome. The resultant peptides are then re-imported into the endoplasmic reticulum (ER) *via* TAP to be loaded onto MHC class I molecules [18], [19]. This pathway, called the “cytosolic cross-presentation pathway,” is believed to have the potential to play pivotal roles in cancer immunotherapy [20]. Although the details of the molecular mechanisms involved in cross-presentation are not yet fully understood, production of interleukin-2 (IL-2) from CD8^+^ T- cells is known to be a hallmark indicating that endocytosed antigens have been cross-presented on MHC class I molecules [18], [21]. For that reason, we first searched for clones that could stimulate IL-2 expression in co-cultures of APCs and antigen-specific CD8^+^ T cells. For this purpose, 10 µg/ml endotoxin-free protein were incubated with DC2.4 cells [22], a mouse dendritic cell line, co-cultured with RF33.70 cells [23], a line of CD8^+^ T-cell hybridoma cells specific for the OVA SIINFEKL epitope (see Methods for the details). Among the proteins tested, only F182A enhanced IL-2 production (Figure 1C–D, and Figure S2 in Supporting Information). Thereafter, eight additional clones with motif contexts similar to F182A were screened for their IL-2 producing ability, and clones F37A and F36C were selected as stimulators of RF33.70 cells (Figure 2A–B, and Figure S3 in Supporting Information). In this *in vitro* experiment, the amount of IL-2 produced from RF33.70 cells by 1 mg/ml OVA was similar to the amount that was produced by 10 µg/ml F37A (Figure S4A, Supporting Information), indicating that F37A is more potent than the native OVA for *in vitro* antigen presentation. Clones F182A and F37A induced IL-2 production when bone marrow-derived dendritic cells were used as the APCs (Figure S4B, Supporting Information). The selected clones (F182A, F37A, F36C) contained triple tandem repeats of the class I epitope. However, it was unclear from our data whether the copy number itself was critical for the induction of IL-2 from the SIINFEKL-specific hybridoma cells, because, the clone G142A contained four copies of the class I epitope but it did not induce IL-2 production (Figure 1C–D). To determine whether all three class I peptide sequences in F37A were indeed important for the T-cell activation, one or more epitope motifs were sequentially substituted with an unrelated sequence, RMFNAPYL (Wilms Tumor 1, MHC class I epitope). In these experiments, we used F37AE2 as a parental clone; it was identical to F37A except for the C-terminal pentapeptide, which was derived from the vector used for constructing the library. While F37AE2 was able to induce IL-2 production from RF33.70 cells, the other seven substituted mutants failed to do so (Figure 2C–D). Thus, all three epitope motifs were necessary for cross-presentation of the artificial antigens. These results suggest that the three epitope motifs within the protein are important for the strong immunogenicity.

### Processing pathway for F37A in APCs

**Figure 3:**
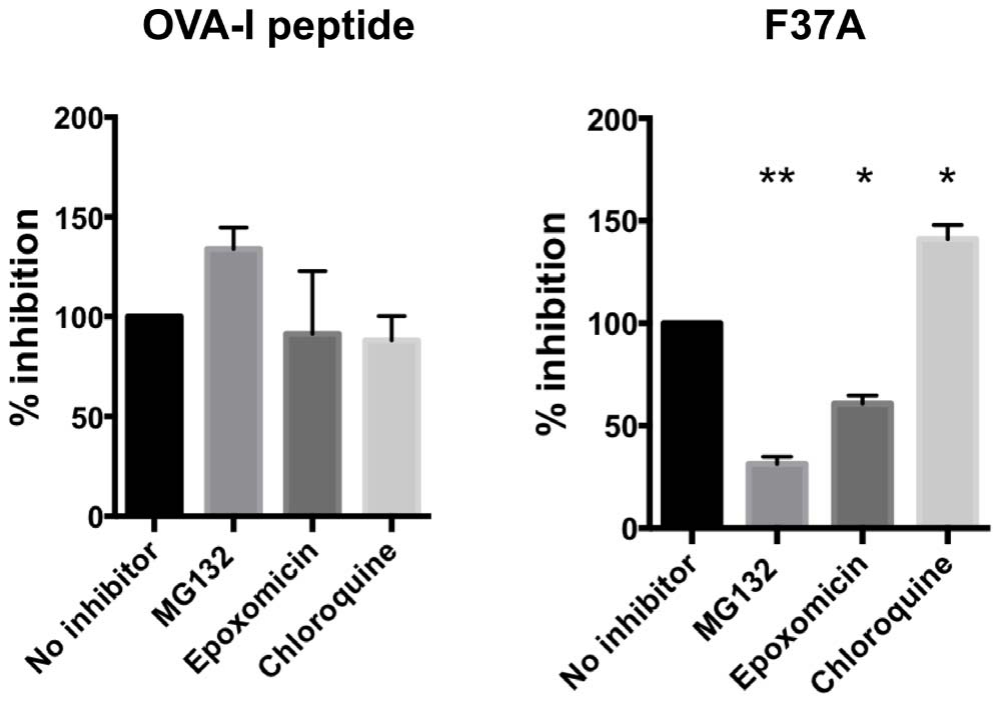
Cross-presentation of F37A was proteasome-dependent. DC2.4 cells were pretreated for 1 h with MG132 (reversible proteasome inhibitor), epoxomicin (irreversible proteasome inhibitor), or chloroquine (lysosome inhibitor) and then co-cultured with RF33.70 cells for 20 h in the continued presence of the inhibitor. Cross-presentation was then determined by measuring the level of IL-2 produced from the RF33.70 cells (as detailed in Materials and Methods). Cross-presentation of F37A was reduced by the proteasome inhibitor, but was increased by chloroquine as compared to the control group (no inhibitor). Each bar indicates percent inhibition in the production of IL-2 concentration compared to that of the control (no inhibitor).*P<0.05, **P<0.01 vs. control.

To confirm that F37A was indeed taken up and processed by APCs, T cell activation assays were performed in the presence of various chemicals known to affect specific components of the antigen processing pathway (Figure 3 and Figure S4C, Supporting Information). First, we evaluated the effect of the proteasome inhibitors MG132 (reversible inhibitor) [22] and epoxomicin (irreversible inhibitor) [24] on antigen presentation. As shown in Figure 3 pretreating APCs with MG132 (20 nM or epoxomicin (50 nM diminished IL-2 release from CTL, suggesting that F37A was processed by proteasome in APCs.

The antigens displayed on MHC class II molecules are processed in the lysosomes, and not by proteasomes. Chloroquine inhibits acidification of endosomes, thereby functioning as an inhibitor of lysosomal antigen degradation. This drug also reportedly increases the cross-presentation of exogenous antigens [3], [25]. Therefore, we next examined the effect of chloroquine on IL-2 production. Results shown in Figure 3 demonstrate that the IL-2 production induced by F37A was augmented by chloroquine, which is consistent with the notion that the amount of antigen entering the cross-presentation pathway is increased by blockade of the class II pathway.

To further elucidate the molecular mechanism underlying the augmented immune response evoked by F37A, we monitored the expression levels of two maturation markers, CD80 and CD86 [26], using flow cytometry to determine whether F37A enhances the maturation of APCs. We found that the addition of F37A did not cause any changes in the expression levels of these co-stimulatory molecules (Figure S5, Supporting Information) suggesting that dendritic cell maturation was not responsible for the enhanced cross-presentation.

**Figure 4:**
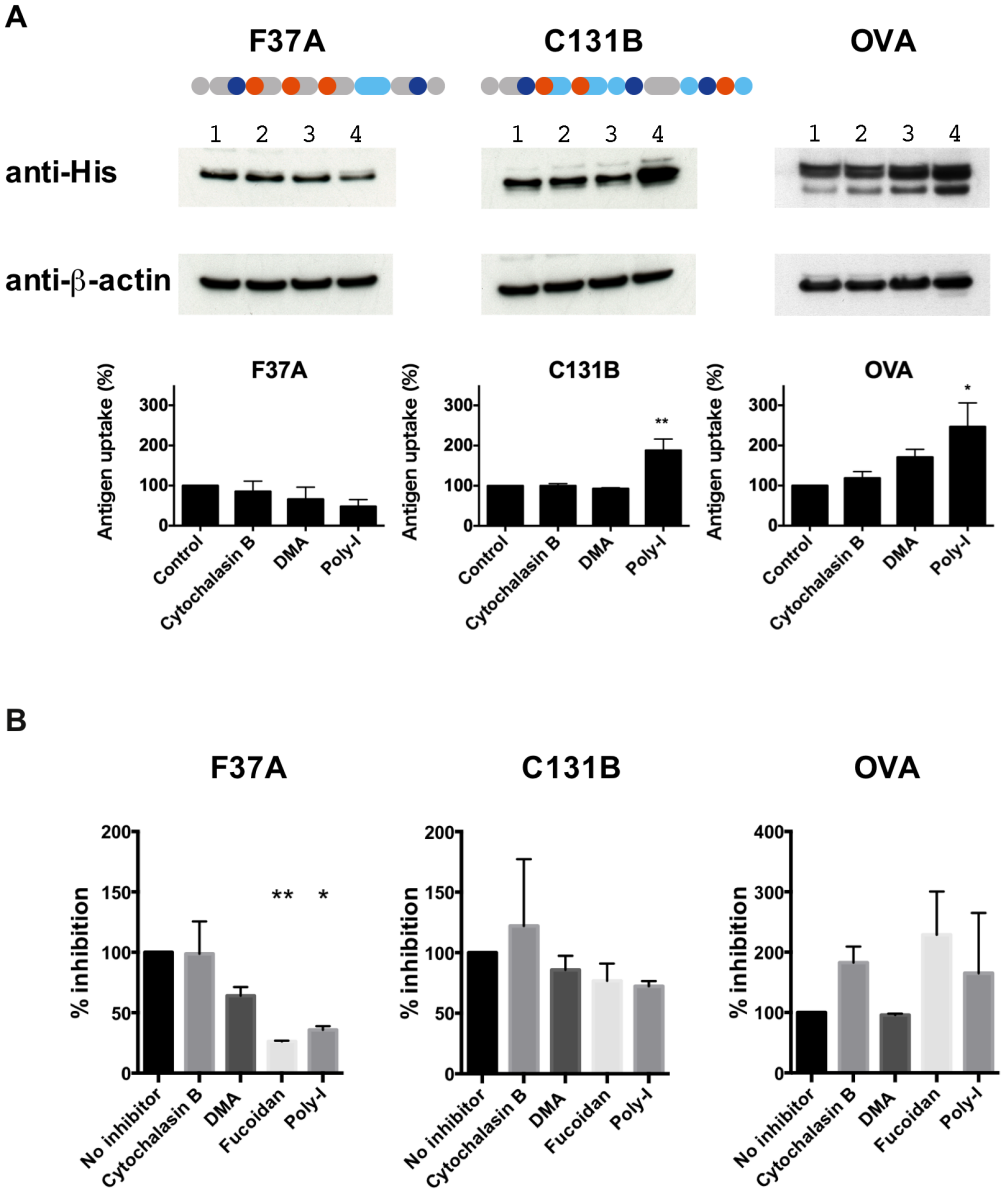
Cross-presentation of F37A was suppressed by Fucoidan and Poly-I, a class A scavenger receptor inhibitor. (**A**) Uptake of antigens by DC2.4 cells and its inhibition by endocytosis inhibitors were measured by Western blot analysis using an anti-His-tag antibody: 1, control; 2, cytochalasin B (phagocytosis inhibitor); 3, DMA (pinocytosis inhibitor); 4, poly-I (class A scavenger receptor inhibitor). Band intensity of the antigen was quantified by densitometry and the intensity was then normalized with respect to that of β-actin. Bars indicate percent changes. Shown at the top are schematic drawings of F37A and C131B; the amino acid sequence of C131B is shown in Figure S2, Supporting Information. (**B**) Cross-presentation of F37A was suppressed by class A scavenger receptor inhibitors (Fucoidan and Poly-I). Each bar indicates percent inhibition in the production of IL-2 concentration compared to that of the control (no inhibitor). *P<0.05, **P<0.01, vs. control.

To determine whether F37A uptake by APCs was augmented, we next incubated APCs with F37A or C131B and compared the amounts of the protein recovered from the cells after washing them. C131B was used as a control because, like F37A, it has three MHC class I epitopes (Figure 4A and Figure S2, Supporting Information). We observed no significant difference in the amount of recovered F37A and C131B (Figure S6A–B, Supporting Information), suggesting that the augmented uptake of F37A by APCs is not responsible for the efficient CTL activation.

There are several pathways that incorporate exogenous antigens within the cell for subsequent cross-presentation; these include macropinocytosis, non-specific phagocytosis and receptor-mediated phagocytosis [27]. Native OVA is phagocytized by APCs after binding to mannose receptors [27]. Because our artificial antigens were synthesized in *E. coli*, they are not glycosylated and so they must be taken up via a mannose receptor-independent pathway. To determine which pathway was responsible for F37A uptake, we performed uptake and CTL stimulation assays in the presence of three endocytosis inhibitors: cytochalasin B, which inhibits phagocytosis; dimethylamiloride (DMA), which inhibits pinocytosis; and polyinosic acid (poly-I), which is a class A scavenger receptor (SRA) inhibitor [27]. The uptake of F37A was moderately suppressed in the presence of poly-I (Figure 4A), suggesting possible involvement, at least partly, of an SRA-based pathway. Interestingly, the uptake of C131B and OVA was augmented by poly-I, though the underlying mechanism remains unknown.

The possible involvement of SRAs in the cross-presentation of F37A was further supported by the observation that IL-2 release induced by F37A was diminished by the addition of fucoidan, another inhibitor of SRA (Figure 4B and Figure S4C, Supporting Information) [28]. SRAs are widely expressed on macrophages and dendritic cells, where they function as pattern recognition receptors. Antigens fused with heat shock proteins are reportedly taken up via SRAs and efficiently cross-presented [29]. Thus, our findings suggest that a pathway involving scavenger receptors could be responsible for the enhanced immunity of F37A. However, further studies will be needed to determine how the antigen is cross-presented in the SRA pathway.

We next used circular dichroism (CD) spectroscopy to examine which secondary structures are present in the artificial proteins (Figure S7, Supporting Information). The ellipticity at 222 nm indicated that all the proteins except F36A contained α-helical structures, which may stem from the encrypted α-helix motif as well as other unintended peptide sequences. With the current data set, however, we are not able to draw any conclusion about the relationship between cross-presentation and secondary structural content. Nevertheless, results from our earlier experiments and the data obtained from the present work, all suggest that encryption of helical structure within designer microgenes leads to artificial proteins that are well expressed in bacterial cells and soluble after purification [8], [10], [30], [31].

### F37A induces OVA-specific CTLs and suppresses OVA-expressing tumor growth *in vivo* without physical adjuvant

**Figure 5:**
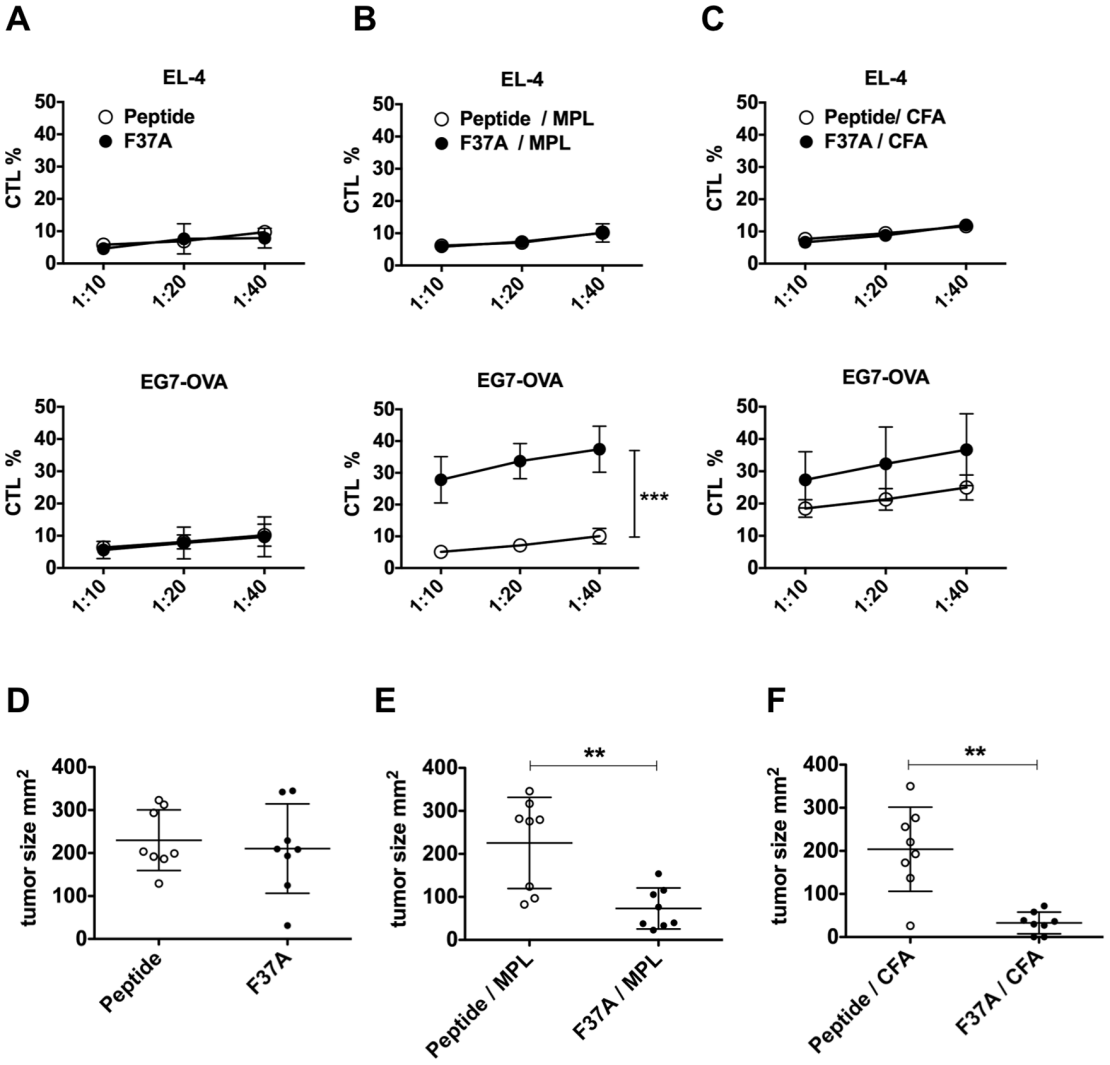
Induction of antigen-specific CTLs and antitumor effects of F37A *in vivo*. (**A–C**) Mice immunized with F37A plus MPL (signal adjuvant) elicited a significant antigen-specific CTL *in vivo*. Mice were intradermally immunized three times with Peptide (free OVA SIINFEKL) or F37A, without and with MPL or CFA. The cytotoxicity of splenic T cells was measured using a standard ^51^Cr releasing assays (CTL assay) against E.G7-OVA cells or OVA-negative EL-4 cells at the target∶effector ratios indicated on the X-axis. Values are expressed as mean ± SD (n = 3). (**D–F**) Immunization with F37A plus MPL or CFA inhibited tumor growth in mice. Following immunization, E.G7-OVA cells (2×10^6^ cells) were inoculated into the backs of mice (n = 8) and tumor volume was monitored. Each dot indicates the tumor volume in an individual mouse 3 weeks after tumor inoculation.

To determine whether F37A can induce T-cell activation *in vivo*, mice were immunized with the short epitopic peptide SIINFEKL (OVA MHC class I peptide; designated here as Peptide) or F37A in combination with MPL or CFA. Macroscopic inflammatory response or change in body weight was not observed in mice injected with F37A and MPL (data not shown). Splenocytes were then isolated from the immunized mice, and induction of antigen-specific CD8^+^ T-cells was evaluated in CTL assays in which EL4 mouse thymoma cells and their derivative, EG7-OVA cells, were used as target cells. EG7-OVA cells are stable EL4 transformants that display OVA epitopes on their MHC class I molecules [32]. As a consequence, EG7-OVA cells are recognized and killed by OVA-specific CTLs, whereas parental EL4 cells are not. By comparing the specific lysis of EL4 and EG7-OVA cells by CTLs prepared from the immunized mice, this assay enables detection of cytolytic activity specific for an OVA epitope in vivo. When no adjuvant was included in the immunization, no CTL response against OVA was detected (Figure 5A). When combined with CFA (signal and physical adjuvants), both the Peptide and F37A elicited OVA-specific CTL responses (Figure 5C). But when antigens were injected with a signal adjuvant (MPL) alone, only F37A elicited specific CTL response; in contrast, the Peptide did not activate any CTL response under those conditions (Figure 5B). This result shows that F37A can evoke cellular immune responses without being combined with an inorganic physical adjuvant.

We next examined the antitumor activity of F37A using an EG7-OVA tumor model. After immunization with F37A, mice were injected with EG7-OVA cells and the subsequent tumor growth was monitored. Notably, F37A showed significant ability to suppress the tumor growth when administered with CFA or MPL, whereas the epitopic peptide (Peptide) did not suppress tumor growth *in vivo*, even though it activated a specific CTL in combination with CFA (Figure 5D–F).

**Figure 6:**
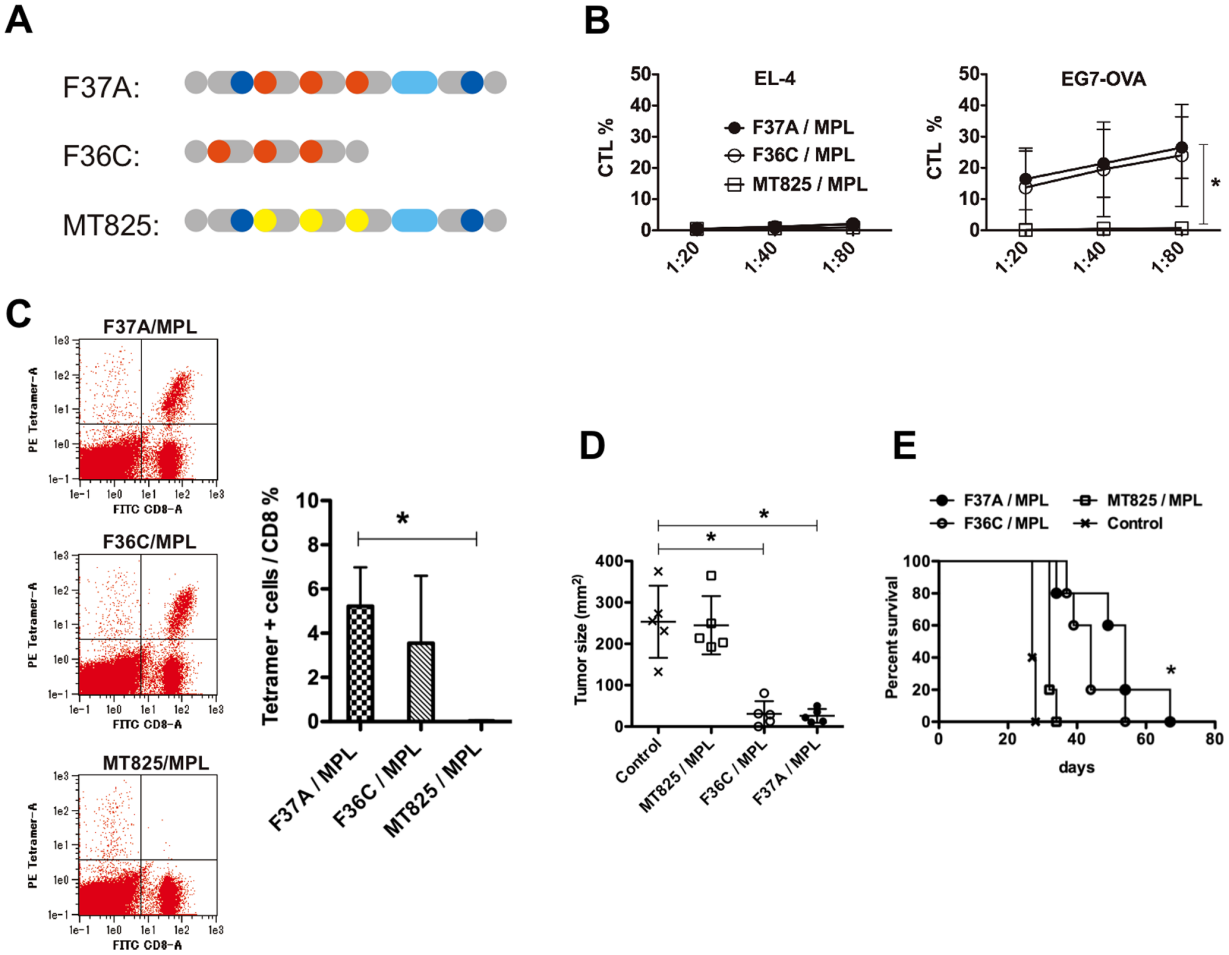
F36C also induced cellular immunity. (**A**) Schematic diagrams of the structures of F37A, F36C and MT825. F36C has a core structure similar to that of F37A, but lacks the class II OVA epitope. (**B**) F36C also induced a significant CTL response, as compared to MT825. Mice were intraperitoneally immunized with antigens plus MPL (n = 3 per condition). CTL responses were assessed as described in Figure 4. (**C**) A representative flow cytometry analysis performed in tetramer assay (left panels). An increase in the OVA-specific CD8 T-cells was observed following immunization with F37A and F36C (right panel). (**D**) Immunization with F36C plus MPL inhibited tumor growth to the same degree as immunization with F37A plus MPL. Bars represent mean ± SD for a group of 5 mice. Each dot indicates the tumor volume in an individual mouse 3-week after tumor inoculation. (**E**) The MHC class II epitope in F37A may function during the effector phase of tumor immunotherapy. Survival plots of mice immunized with F37A/MPL, F36C/MPL, or MT825/MPL. Mice immunized with either F37A/MPL or F36C/MPL survived longer than the unimmunized control group. Moreover, mice immunized with F37A survived longer than those immunized with F36C (n = 8 mice per group).

Finally, we examined the effect of MHC class II epitope (OVA-II), encrypted in the designer microgenes (Figure 1), on the antitumor capability of F37A. After confirming that F37A elicited humoral immunity against OVA (Figure S8, Supporting Information), we compared the antitumor activities of F37A and F36C, which has a structure similar to that of F37A but lacks the class II OVA epitope (Figure 6A). Because F36C retains all the class I epitopes, it induced cellular immunity, just as F37A did (Figure 6B). On the other hand, the control protein MT825, within which all OVA class I epitopes were replaced with unrelated sequences, did not induce functional CTLs. MHC tetramer assay was then performed to detect the OVA-I (class I epitope, SIINFEKL)-specific CTL cells in immunized mice. Prior to the tetramer assay, antigen-reactive T-cells were expanded *in vitro* as described in the Methods, which makes the assay qualitative. From this assay, we confirmed the presence of SIINFEKL-specific CD8^+^ T cells (tetramer-positive cells) in F37A/MPL- and F36C/MPL-immunized mice (Figure 6C). Moreover, both F37A and F36C suppressed the growth of tumors by three weeks after the inoculation (Figure 6D). Although the tumor sizes increased during the rest of their lifespan (Figure S9, Supporting Information), the survival periods of mice immunized with F37A were prolonged compared to the ones from the unimmunized control group (Figure 6E), indicating efficacy of the vaccine.

CD4^+^ T-cells play a cardinal role both in the antibody production and the activation and expansion of CD8^+^ T-cells. This is known as “CD4^+^ T-cell help” [33] and is a prerequisite for CD8^+^ T-cell priming and maintenance of CD8^+^ T-cell memory. It is also required for the post-priming phase of CD8^+^ T-cell that occurs at the tumor site. An optimal CD4^+^ T-cell response can augment the accumulation of CD8^+^ T-cells within a tumor and promote the expansion, trafficking and differentiation of tumor-specific CD8^+^ T-cells, all of which enhance antitumor immunity. Many antibodies recognize a conformational epitope with a specific three-dimensional shape and its structure. However, the induction of anti-OVA antibody production by F37A immunization in mice suggested that OVA-II (class II epitope, ISQAVHAAHAEINEAGR) derived from F37A antigen functions as a linear epitope and may also be presented with class II molecules. Therefore, CD4^+^ T-cells, which recognizes the OVA-II derived from F37A, may be involved in the antitumor effects during the effector phase of cancer immunity via CD4^+^ T-cell help.

When peptides are used as vaccines, co-administration of oil emulsion adjuvant (for instance, IFA) is essential to fully induce an immune reaction [34]. For instance, the cervical cancer treatment *Cervarix* includes the adjuvant AS04, which is a complex of monophosphoryl lipid A and aluminum salt [6]. However, the poor definition of physical adjuvants has hindered the development of vaccines for cancer, AIDS and malaria, among others, and only a small number of adjuvants have been approved for use in vaccines [4]–[7]. In addition, one recent study suggested that the local inflammation caused by IFA may congregate cytotoxic CTLs at the vaccination site, thereby hindering recruitment of CTLs to the tumor site [35]. And since the subcutaneously injected protein antigens are recognized as exogenous antigens by APCs, antigenic epitopes are mainly presented on class II molecules, leading to a humoral immune response [36]. Although invoked cellular immune responses are important for cancer immunotherapy, a safe and efficient adjuvant system that could induce cellular immunity still remains to be established [37]. Our artificial antigens are not dependent on an oil-based adjuvant (physical adjuvant) to induce cellular immunity, which reduces the chances of occurring unwanted local inflammation at the injection sites. Furthermore, they have the potential to evolve into adjuvant-free (adjuvant-included) polypeptide vaccines through rational addition of peptidic motifs that can signal Toll-like receptors. It has been reported that TLR-4 signaling pathway can be activated by Toll-like receptor 4 agonist peptides [38]. In the next step, we have a plan to incorporate such a TLR-4 agonist motif into the artificial antigen F37A.

In summary, we have succeeded in preparing an artificial protein antigen that can evoke cellular immune responses without being combined with an inorganic physical adjuvant. The system described may be further developed for the creation of physical adjuvant-free vaccines, perhaps for use against AIDS, malaria or cancer, ailments in which cellular immunity plays a critical role in treatment.

## Materials and Methods

### Ethics Statement

All animal procedures were performed as written in a protocol reviewed and approved by the Institutional Animal Care and Use Committee at Jikei University (protocol #22-010C2, #22-038C2) before the beginning of the study. The major causes of death or moribundity in mice were transplanted tumors. Mice were monitored every 2–3 days and tumors were measured using digital calipers until the tumor size was ≥400 mm^2^, at which time the humane endpoint was met and mice were anaesthetized by intraperitoneal injection of sodium pentobarbital solution (50 mg/kg), then euthanized by cervical dislocation. If the mouse appeared moribund (cachexia, weight loss, and abdominal swelling due to ascites formation), indicating low probability of surviving for greater than 24 hours, it was also euthanized.

## Materials

Antibodies for flow cytometry were purchased from eBioscience and Biolegend. The OVA MHC class I epitope peptide OVA259–265 (OVA-I, SIINFEKL) was purchased from MBL, Japan. RF33.70 and DC2.4 cells were kindly provided by K.L. Rock (University of Massachusetts Medical School, USA). EL-4 thymoma and E.G7-OVA cells (OVA-transfected clone of EL-4) were purchased from the ATCC. Female C57BL/6 (B6; H-2b) mice were purchased from The Sankyo Labo Service, Japan.

### Construction of Artificial Proteins

The method used to construct artificial proteins has been described in detail elsewhere [8]–[11]. In brief, polymers of microgenes MG-2101 and MG-6101 (Figure 1A) were prepared using the microgene polymerization reaction (MPR) method, with which insertions or deletions of nucleotides randomly occur at junctions between microgene units, endowing the translated products with combinatorial molecular diversity [8]. The resultant microgene polymers were ligated into the expression vectors pKS601, pKS603 and pKS605, each of which can translate one of three coding frames of the microgene polymers as an N-terminal His-tagged fusion protein. Recombinant proteins were expressed in *E. coli* XL1Blue cells (Stratagene) and were purified using TALON resin (Clontech). Protein purity was assessed using SDS-PAGE.

### Endotoxin removal

We used the method of Reichelt [39] to efficiently remove contaminating endotoxins from the recombinant proteins. Mn TALON agarose-bound recombinant proteins were extensively washed in Triton X-114 buffer (1% (v/v) Triton X-114 (Sigma #6858), 8 M Urea, 50 mM NaH_2_PO4, 100 mM NaCl, 15 mM imidazole, pH 7.0) at 4°C, after which the proteins were eluted with imidazole and dialyzed against glycine-HCl buffer (pH 3.0). Endotoxin-free native OVA protein was obtained from chicken egg white ovalbumin (Sigma, A-5503) using Triton X-114 as described [40]. Endotoxin levels were monitored by a limulus amoebocyte lysate (LAL) assay using an Endosafe-PTS device (Charles River Laboratories) after diluting the recombinant proteins 1∶40 in endotoxin-free water. We confirmed that the endotoxin levels in the antigens used in this work were less than 0.5 EU/mg.

### *In vitro* antigen presentation assay

DC2.4 cells (dendritic cell line) were seeded onto a 96-well flat-bottomed culture plate to a density of 1×10^4^ cells/well in AIM-V medium (Invitrogen) and cultured overnight, after which the cells were incubated overnight with antigens. Each well was then treated with mitomycin C (10 µg/ml) for 30 min and washed twice with PBS before co-culturing the DC2.4 cells with RF33.70 OVA-SIINFEKL specific T-T hybridoma cells (1×10^5^ cells/well) against OVA+H-2 Kb for 20 h. We assessed the response of the stimulated RF33.70 cells based on the amount of IL-2 released into an aliquot of culture medium (100 µl), which was measured using a murine IL-2 ELISA kit (BD Biosciences). In some assays, live bone marrow-derived dendritic cells (10^5^ cells/well) were used.

### Pharmacological inhibition assay

DC2.4 cells (4×10^4^ cells/well) were pretreated for 1 h with the inhibitors before the addition of antigen. The following inhibitors were used for this assay: MG132 (Peptide Institute, Inc. 3175-v, 20 nM), epoxomicin (Sigma E3652, 50 nM), chloroquine (Sigma C6628, 10 µM), cytochalasin B (Sigma C6762, 100 nM), dimethylamiloride (DMA, Sigma A4652, 100 nM), poly-I (Sigma P4154, 100 µg/ml) and fucoidan (Sigma F5631, 100 µg/ml). Cells were then further incubated with one of the following antigens at 37°C for 4 h in the continued presence of the inhibitor: OVA-I (20 ng/ml), F37A (10 µg/ml), C131B (10 µg/ml) or OVA (10 mg/ml). Then DC2.4 cells were co-cultured with RF33.70 cells (4×10^4^ cells/well) for 20 h and IL-2 production was measured as described above. We confirmed that the activation response of RF33.70 cells used in the pharmacological inhibition assay was similar for the inhibitor-treated and untreated DCs using OVA-I peptide as an assay control.

Antigen taken up by DC2.4 cells was determined by Western blot analysis. DC2.4 cells were separately pretreated for 30 min with the following inhibitors: 100 µM cytochalasin B, 500 µM DMA, and 50 µg/ml poly-I. Cells were incubated further with the antigen for 30 min at 37°C in the continued presence of the inhibitors and then washed three times with PBS. After the preparation of cell lysates in RIPA buffer (50 mM Tris-HCl [pH 7.6], 150 mM NaCl, 1% Nonidet-P40, 0.5% sodium deoxy cholate, protease inhibitor cocktail and 0.1% SDS), aliquots (30 µg) of proteins derived from control and treated cells were subjected to SDS-PAGE and analyzed by Western blotting using an anti-his-tag antibody (MBL, D291-3, clone OGHis). The relative amounts of artificial antigen taken up by the dendritic cells were determined by quantifying the band intensity using densitometry and then normalizing the intensity to that of β-actin.

### *In vivo* immunization

Mice were immunized with Peptide (SIINFEKL), F37A (181 aa), F36C (120 aa) or MT825 (186 aa). Each mouse was injected intradermally (into the hind footpad) or intraperitoneally three times with 100 µg of antigen (103.8 nmol for Peptide; 2.3 nmol for OVA; 4.9 nmol for F37A; 7.2 nmol for F36C; 4.8 nmol for MT825) with or without adjuvants at 14-day intervals. Monophosphoryl lipid A (MPL) (10 µg per mouse, Sigma L6895) and complete Freunds's adjuvant (CFA) were used as adjuvants. CFA was obtained by mixing 5 mg/ml heat-killed *M. tuberculosis* H37Ra into incomplete Freund's adjuvant (DIFCO).

### *In vitro* stimulation of T cells

For *in vitro* stimulation, splenocytes (3×10^6^) prepared from immunized mice were incubated for 5 days with 1×10^6^ irradiated (100 Gy) E.G7-OVA cells in 1 ml of RPMI 1640 medium supplemented with 10% FBS and IL-2 (20 ng/ml) in a 24-well culture plate. Tetramer assay and CTL assay were then carried out on days 3 and 5, respectively.

### CTL assay

Target cells (1×10^6^) were first labeled with 5.9 MBq of ^51^CrNa_2_CrO_4_ (MP Bio, #62015) in 20 µl of FBS for 1 h at 37°C. The cells were then washed three times with complete medium, and 1×10^4^ of the ^51^Cr-labeled targets were added to each well in a 96-well U-bottom plate. Effector cells (splenocytes) were serially diluted and assayed in triplicate at the indicated target-to-effector cell ratios (T/E). After incubation for 5 h at 37°C, the supernatants (30 µl) were collected, and the amount of radioactivity was counted in a gamma counter (Packard). The percent specific lysis was determined as 100× [(release by CTL – spontaneous release)/(maximum release – spontaneous release)]. Maximum release was determined by the addition of 100 µl of 1% Triton X-100. In all experiments, the spontaneous release in the absence of effector cells was less than 15% of the maximum release.

### Tetramer assay

The frequency of OVA-specific CD8^+^ effector T-cells was determined by tetramer staining *ex vivo*. *In vitro*-stimulated splenocytes were stained with T-select H-2 Kb OVA Tetramer-SIINFEKL-PE (MBL, Japan) for 20 min at 4°C according to the manufacturer's protocol. Fluorescence was analyzed using a MACSQuant analyzer (Miltenyi Biotech).

### Tumor experiments

For prophylactic tumor experiments, groups of 5 or 8 mice were immunized with antigens. Fourteen days after the last boost, the mice were subcutaneously injected with 2×10^6^ E.G7-OVA cells in a total volume of 300 µl into the lower back. Tumor sizes were measured as the product of two perpendicular tumor diameters.

### Statistical analysis

Statistical analysis was performed using Prism 5 (GraphPad). Data used for plotting were mean ± SD (*P<0.05, **P<0.01, ***P<0.001). One-way ANOVA with Dunnett's multiple comparison test was used for Figures 3, 4, 6C and S6C (Supporting Information). Two-way ANOVA with Bonferroni's post-hoc test was used for Figures 5A–C and 6B. The Mann Whitney test was used for Figure 5D–F. The Kruskal-Wallis test with Dunn's multiple comparison test was used for Figure 6D. For comparison of the Kaplan-Meier survival curves in Figure 6E, the log rank test was applied. Unpaired t-tests were used for Figure S8.

## Supporting Information

Figure S1 **Structures of model antigen OVA and microgenes.** (**A**) Amino acid sequence of OVA (GenBank AAB59956.1) showing the MHC class I (red) and MHC class II (blue) epitopes. (**B**) Microgene primers and microgene design. Class I epitope (red), class II epitope (blue), a β-sheet motif (brown) and an α-helix motif (sky blue) were coded in three different reading frames of the microgenes.
(TIF)

Figure S2 **Primary structures of the artificial proteins used for the initial screening.** The class I epitope (red), class II epitope (blue), β-sheet motif (brown) and α-helix motif (sky blue) are shown.
(TIF)

Figure S3 **Primary structures of the artificial proteins used in the second screening.** The class I epitope (red), class II epitope (blue) and α-helix motif (sky blue) are shown.
(TIF)

Figure S4 ***In vitro*** **antigen presentation assay.** (**A**) In an *in vitro* antigen presentation assay, native OVA exhibited antigen presenting function only when DC2.4 cells were treated with a high concentration (1 mg/ml) of antigen. (**B**) Mouse bone marrow-derived dendritic cells prepared from monocytes by inducing differentiation with GM-CSF efficiently presented F182A and F37A on MHC class I molecules. OVA-specific T cell hybridoma (RF33.70) cells were cultured with bone marrow-derived dendritic cells in the presence of 10 µg/ml of the indicated antigen. Data shown are mean IL-2 concentration ± SD (n = 3). (**C**) *in vitro* antigen presentation assay. DC2.4 cells were treated with the indicated antigen for 4 h and then co-cultured with RF33.70 cells for 20 h in the absence of the inhibitor. IL-2 production from RF33.70 cells under this condition was used as a control (no inhibitor) for the pharmacological inhibition assay in Figures 3 and 4B.
(TIF)

Figure S5 **Artificial antigen F37A does not induce dendritic cell maturation.** Bone marrow-derived dendritic cells were stimulated with 10 µg/ml F37A (red line), 10 µg/ml lipopolysaccharide (LPS; green line) or no protein (blue line) for 24 h, following which they were stained and analyzed by flow cytometry for the expression of maturation markers CD80 and CD86.
(TIF)

Figure S6 **DC2.4** **cells take up similar amounts of F37A and C131B.** (**A**) Different amounts (100 ng, 50 ng, 10 ng and 5 ng) of his-tagged antigen were subjected to Western blot analysis using an anti-his-tag antibody (MBL Japan, clone; OGHis). A linear relationship was found between the intensity of the chemiluminescent signal and the amount of antigen used; this was used as a standard curve (data not shown). (**B**) DC2.4 cells were incubated for 30 min in the presence of 10 µg/ml C131B or F37A, after which whole cell lysates were prepared in RIPA lysis buffer (50 mM Tris·HCl [pH 7.4], 150 mM NaCl, 1% Triton X-100 and proteinase inhibitors). Protein concentrations were then determined using BCA assays, and 30-µg aliquots were resolved using 4–12% SDS-PAGE. Signals from the Western blot were compared to the standard curve to estimate the antigen content in the DC2.4 cells.
(TIF)

Figure S7 **Far UV circular dichroism (CD) spectra of artificial proteins.** Analysis of CD spectra of F37A, F182A and F36C showed to contain secondary structure that was not observed in native OVA. CD spectra of native OVA and artificial proteins F182C, F37C and F36B were typical of proteins forming α-helical structures. F182B and F36A showed a random coil structure. Data were collected on a JASCO J-725 at 25°C by accumulating five scans. Proteins samples (10 µM) used for the CD analysis were prepared in 10 mM phosphate buffer (pH 5.0).
(TIF)

Figure S8 **F37A induced both cellular and humoral immunity.** Mice were intradermally immunized with the indicated antigens, with or without adjuvants (n = 3 per condition). Serum was then collected from the immunized mice, and OVA-specific antibody production was determined by ELISA using OVA as an antigen. Antibody production was observed in the mice group immunized with F37A (plus MPL or CFA), but not in the group immunized with Peptide.
(TIF)

Figure S9 **Tumor growth in mice immunized with F37A and F36C.** Mice were intraperitoneally immunized with the indicated antigens plus MPL (n = 5 per condition). Following immunization, E.G7-OVA cells (2×10^6^ cells) were inoculated into the back of each mouse and growth of the tumor was monitored by measuring the tumor volume. Control mice were not immunized with any antigen.
(TIF)
