# Supplementary figures and images for: Combinatorial Contextualization of Peptidic Epitopes for Enhanced Cellular Immunity
Source: PLoS One. 2014 Oct 24;9(10):e110425. doi: 10.1371/journal.pone.0110425 (PMC4208766; doi:10.1371/journal.pone.0110425)

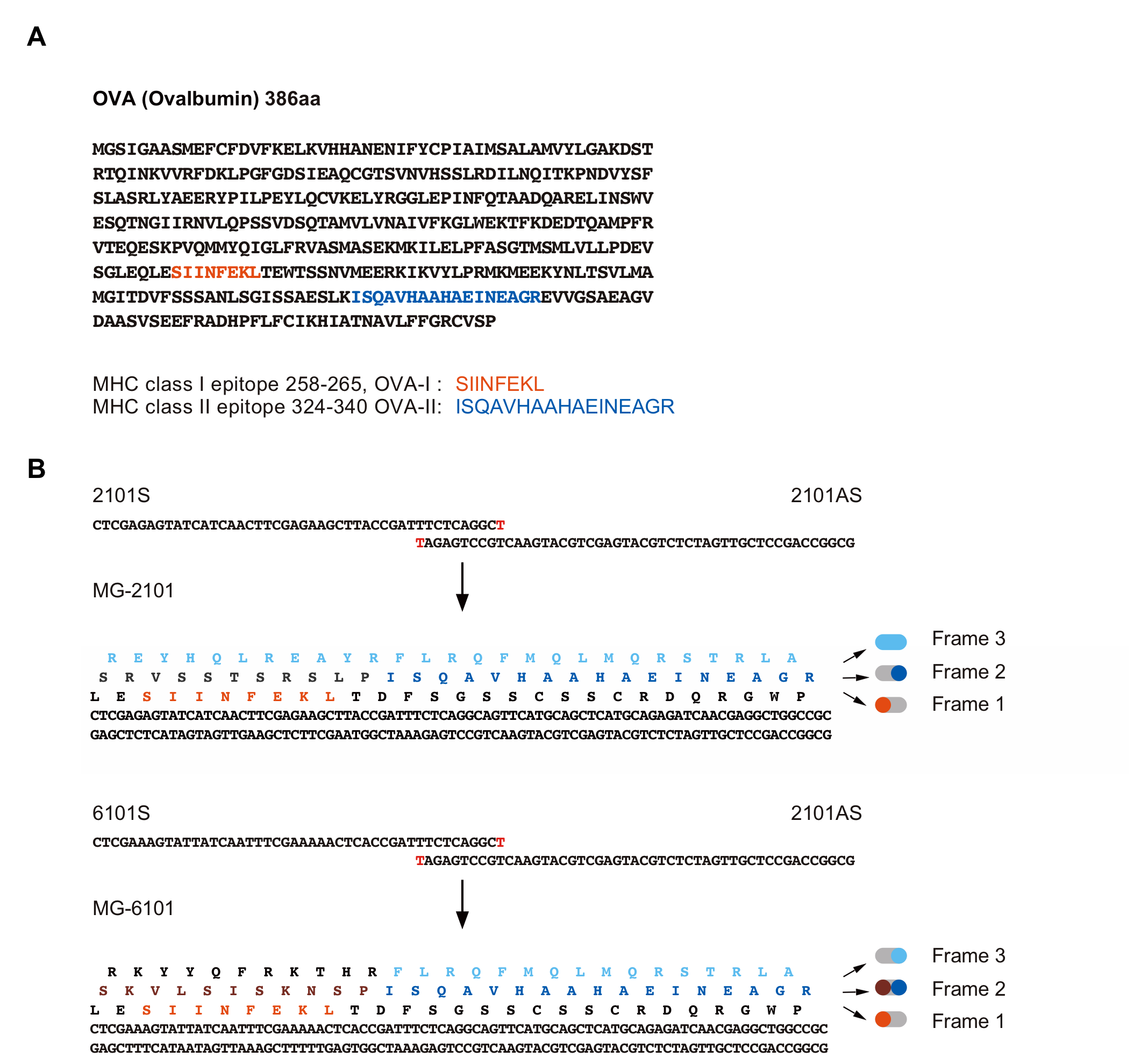

Supplement: Figure S1 — Structures of model antigen OVA and microgenes. (A) Amino acid sequence of OVA (GenBank AAB59956.1) showing the MHC class I (red) and MHC class II (blue) epitopes. (B) Microgene primers and microgene design. Class I epitope (red), class II epitope (blue), a β-sheet motif (brown) and an α-helix motif (sky blue) were coded in three different reading frames of the microgenes. (TIF) [file pone.0110425.s001.tif]

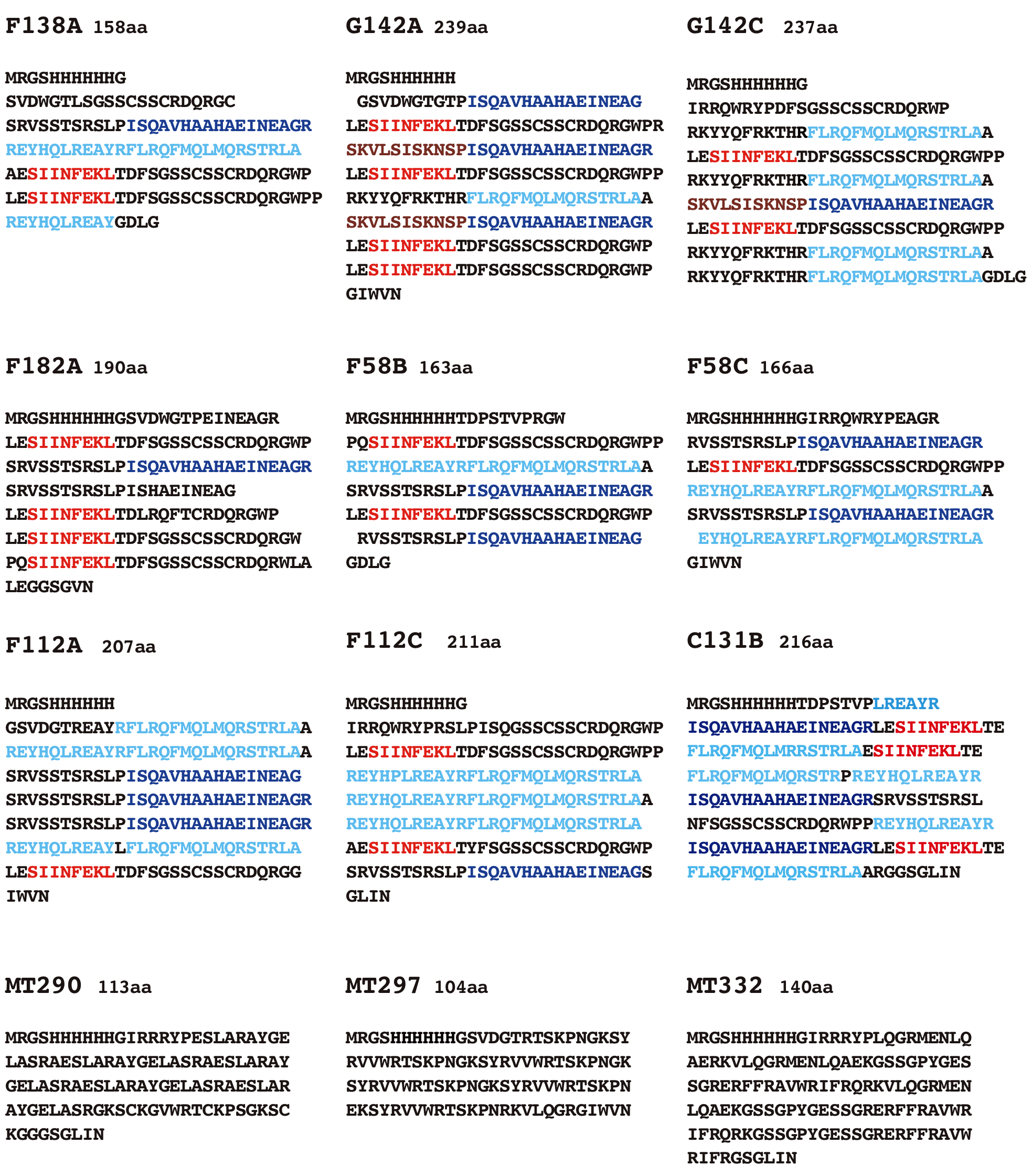

Supplement: Figure S2 — Primary structures of the artificial proteins used for the initial screening. The class I epitope (red), class II epitope (blue), β-sheet motif (brown) and α-helix motif (sky blue) are shown. (TIF) [file pone.0110425.s002.tif]

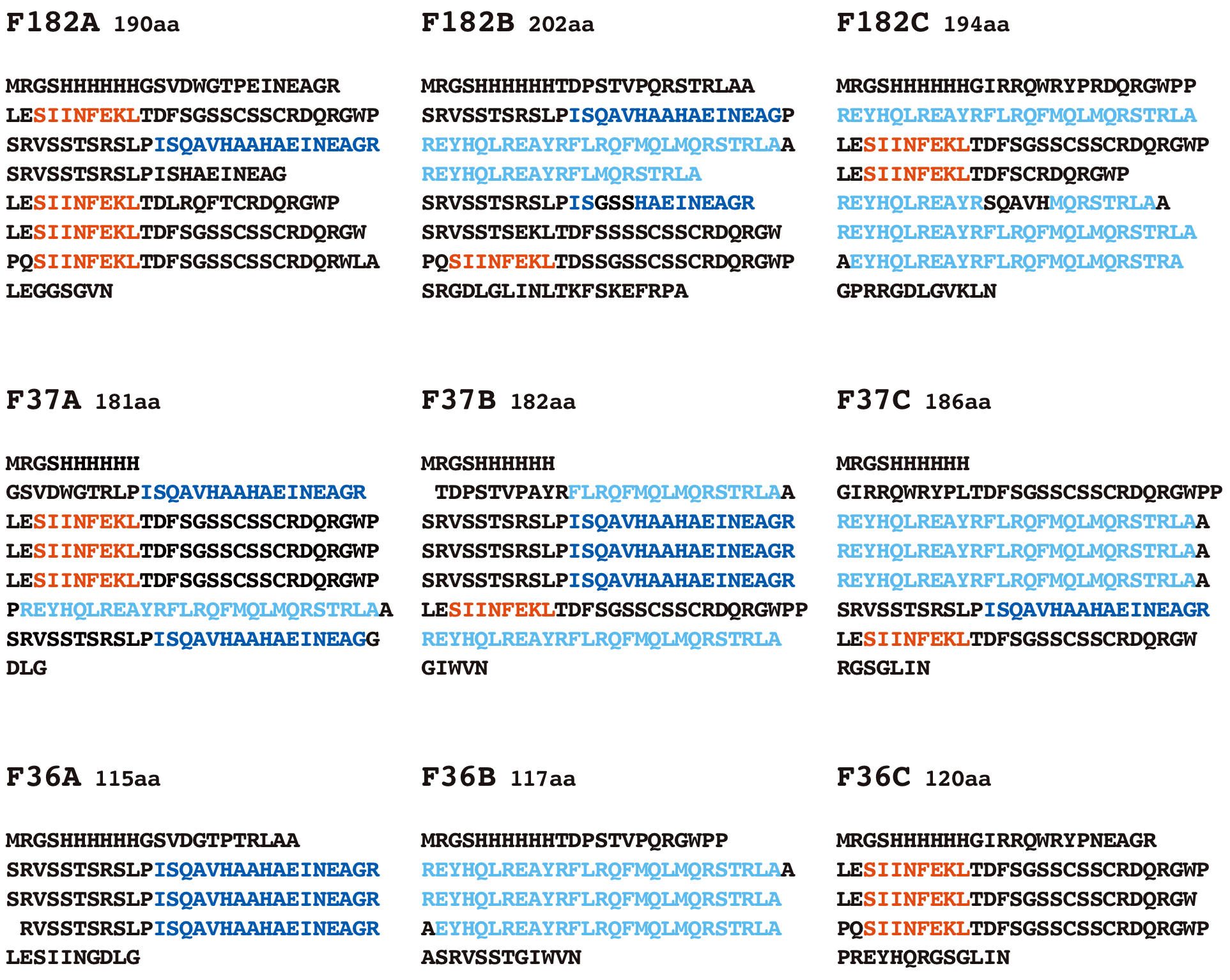

Supplement: Figure S3 — Primary structures of the artificial proteins used in the second screening. The class I epitope (red), class II epitope (blue) and α-helix motif (sky blue) are shown. (TIF) [file pone.0110425.s003.tif]

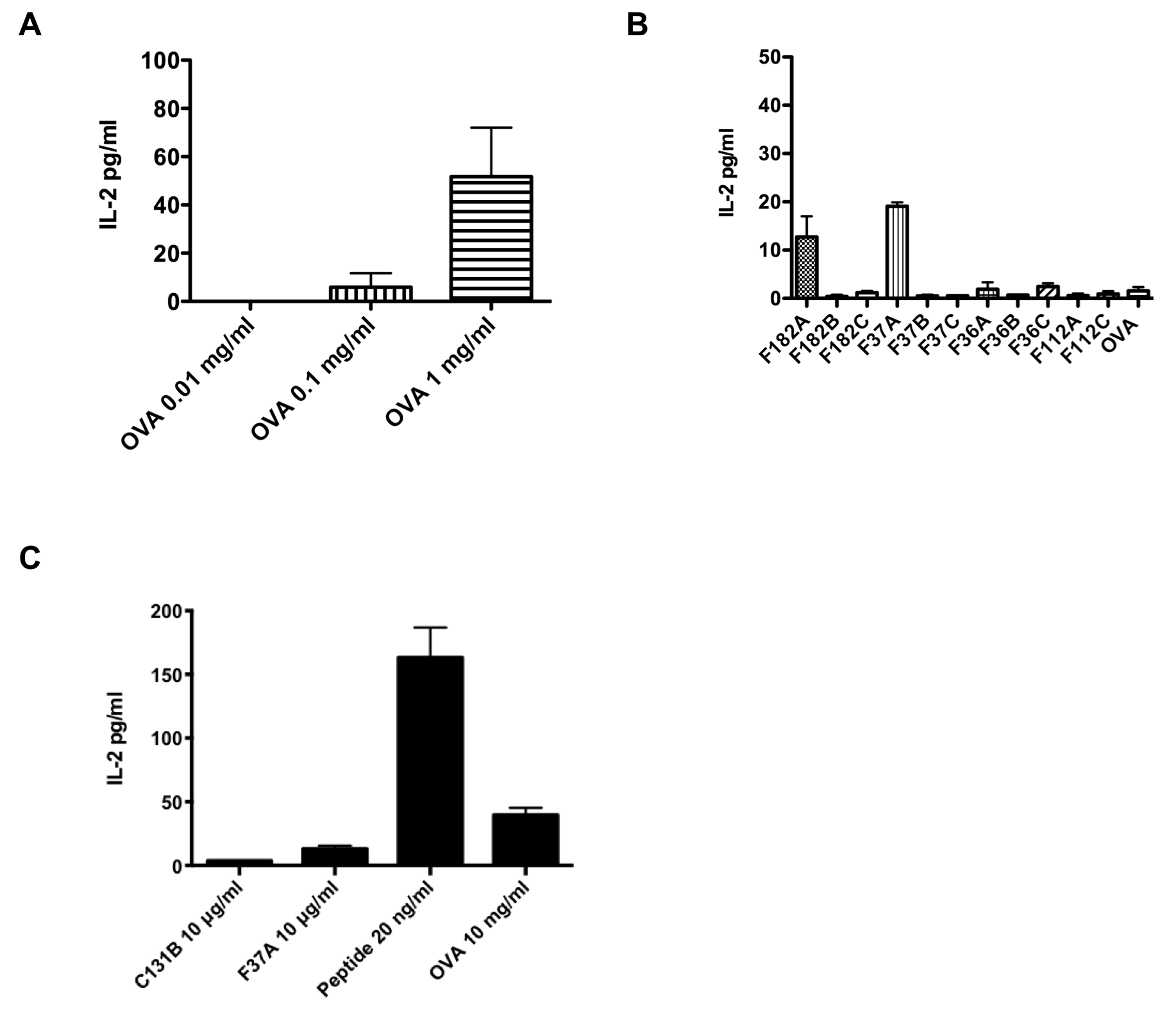

Supplement: Figure S4 — In vitro antigen presentation assay. (A) In an in vitro antigen presentation assay, native OVA exhibited antigen presenting function only when DC2.4 cells were treated with a high concentration (1 mg/ml) of antigen. (B) Mouse bone marrow-derived dendritic cells prepared from monocytes by inducing differentiation with GM-CSF efficiently presented F182A and F37A on MHC class I molecules. OVA-specific T cell hybridoma (RF33.70) cells were cultured with bone marrow-derived dendritic cells in the presence of 10 µg/ml of the indicated antigen. Data shown are mean IL-2 concentration ± SD (n = 3). (C) in vitro antigen presentation assay. DC2.4 cells were treated with the indicated antigen for 4 h and then co-cultured with RF33.70 cells for 20 h in the absence of the inhibitor. IL-2 production from RF33.70 cells under this condition was used as a control (no inhibitor) for the pharmacological inhibition assay in Figures 3 and 4B. (TIF) [file pone.0110425.s004.tif]

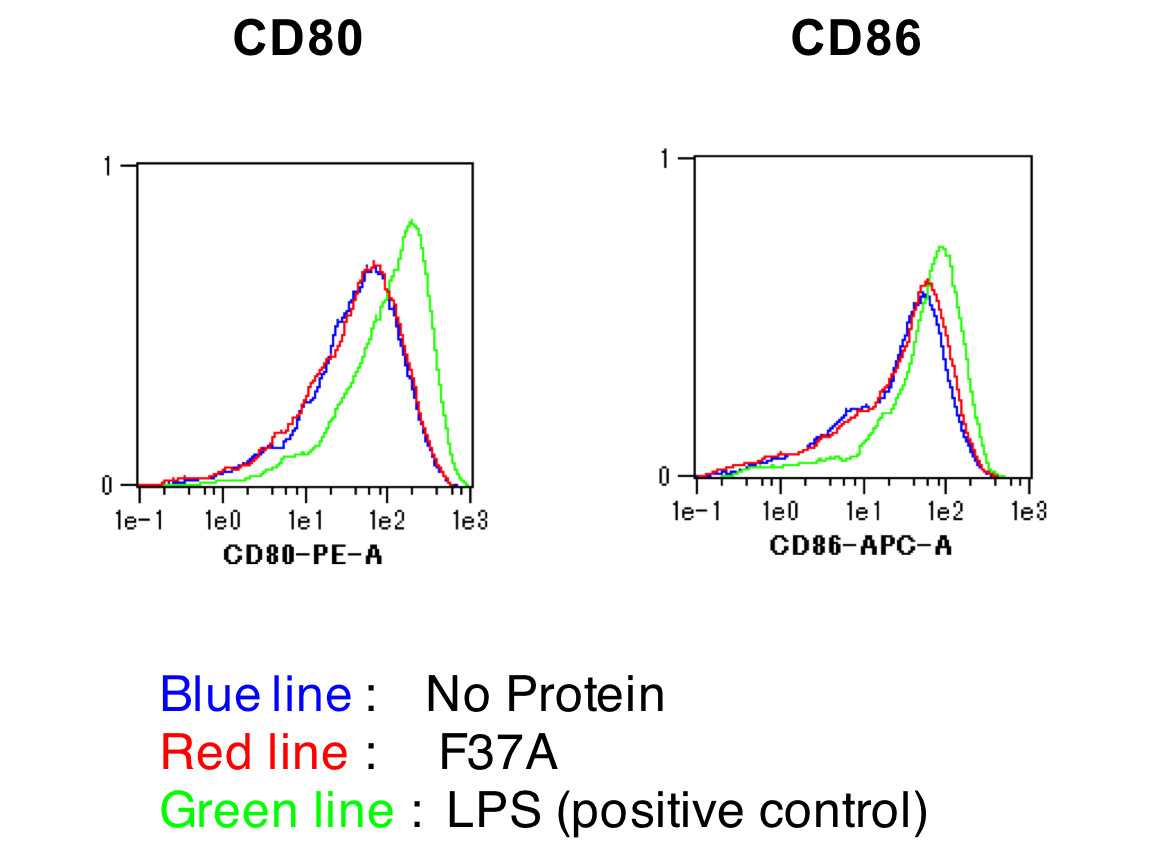

Supplement: Figure S5 — Artificial antigen F37A does not induce dendritic cell maturation. Bone marrow-derived dendritic cells were stimulated with 10 µg/ml F37A (red line), 10 µg/ml lipopolysaccharide (LPS; green line) or no protein (blue line) for 24 h, following which they were stained and analyzed by flow cytometry for the expression of maturation markers CD80 and CD86. (TIF) [file pone.0110425.s005.tif]

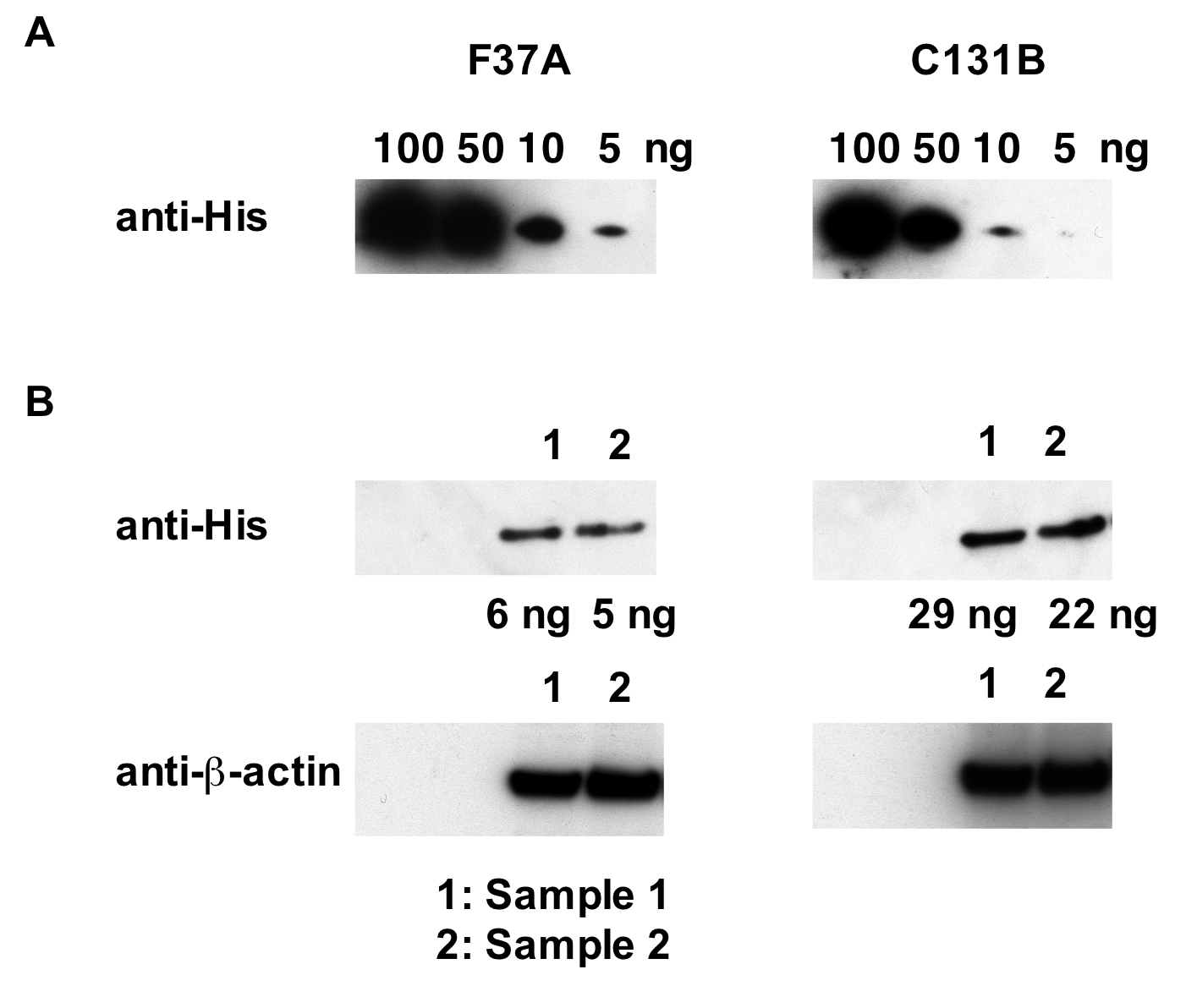

Supplement: Figure S6 — DC2.4 cells take up similar amounts of F37A and C131B. (A) Different amounts (100 ng, 50 ng, 10 ng and 5 ng) of his-tagged antigen were subjected to Western blot analysis using an anti-his-tag antibody (MBL Japan, clone; OGHis). A linear relationship was found between the intensity of the chemiluminescent signal and the amount of antigen used; this was used as a standard curve (data not shown). (B) DC2.4 cells were incubated for 30 min in the presence of 10 µg/ml C131B or F37A, after which whole cell lysates were prepared in RIPA lysis buffer (50 mM Tris·HCl [pH 7.4], 150 mM NaCl, 1% Triton X-100 and proteinase inhibitors). Protein concentrations were then determined using BCA assays, and 30-µg aliquots were resolved using 4–12% SDS-PAGE. Signals from the Western blot were compared to the standard curve to estimate the antigen content in the DC2.4 cells. (TIF) [file pone.0110425.s006.tif]

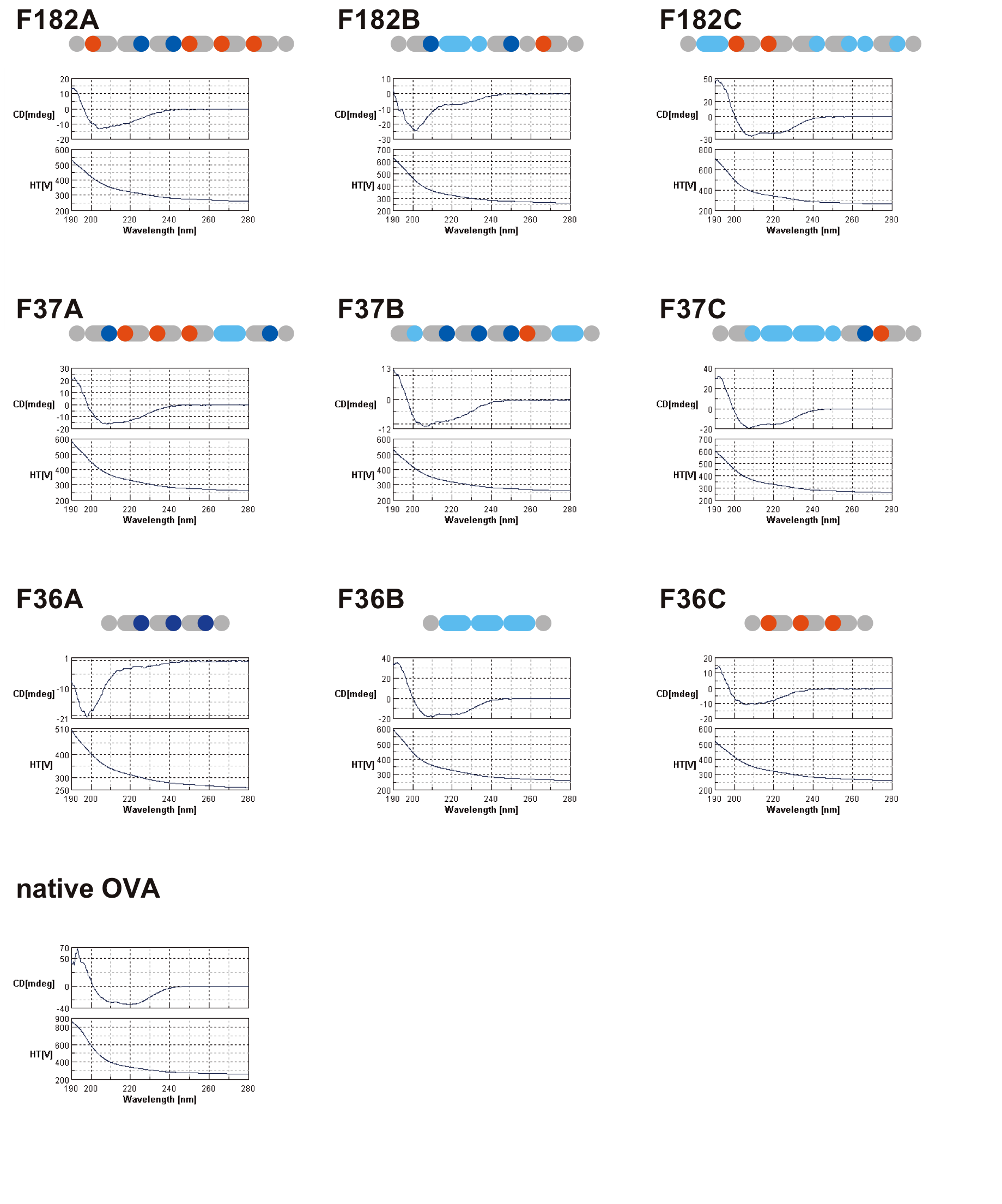

Supplement: Figure S7 — Far UV circular dichroism (CD) spectra of artificial proteins. Analysis of CD spectra of F37A, F182A and F36C showed to contain secondary structure that was not observed in native OVA. CD spectra of native OVA and artificial proteins F182C, F37C and F36B were typical of proteins forming α-helical structures. F182B and F36A showed a random coil structure. Data were collected on a JASCO J-725 at 25°C by accumulating five scans. Proteins samples (10 µM) used for the CD analysis were prepared in 10 mM phosphate buffer (pH 5.0). (TIF) [file pone.0110425.s007.tif]

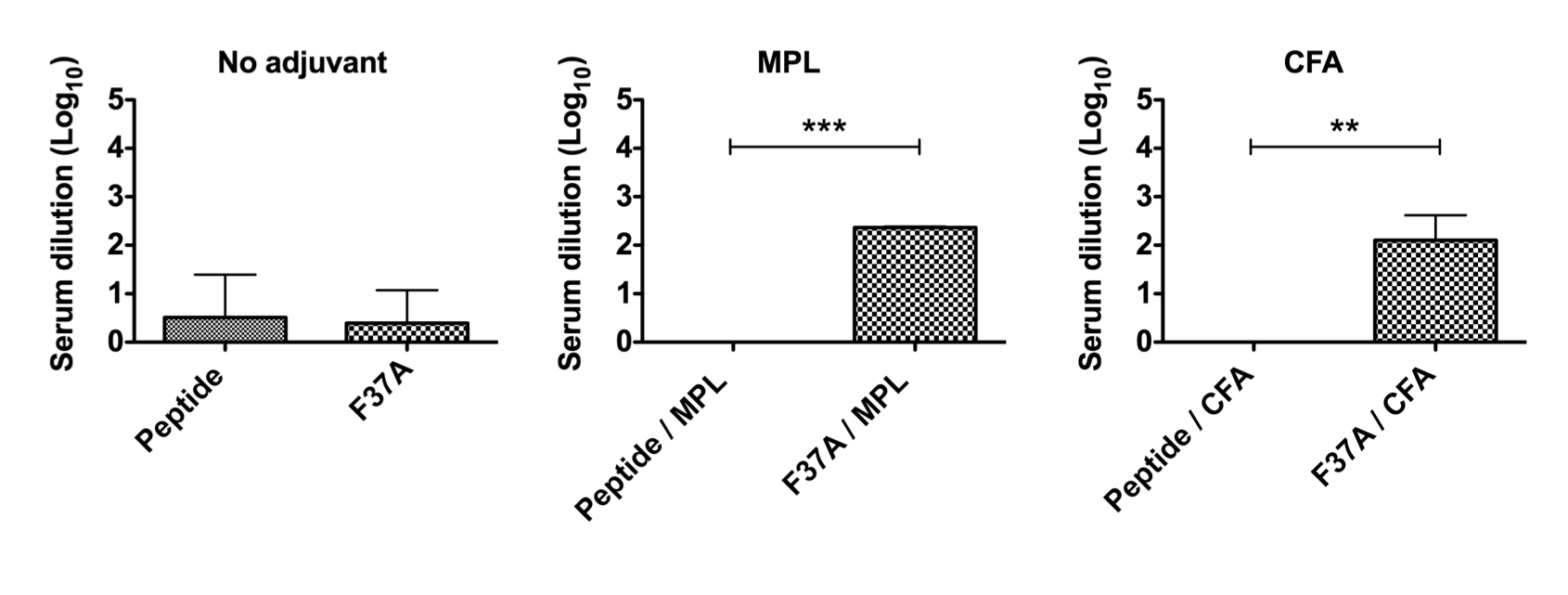

Supplement: Figure S8 — F37A induced both cellular and humoral immunity. Mice were intradermally immunized with the indicated antigens, with or without adjuvants (n = 3 per condition). Serum was then collected from the immunized mice, and OVA-specific antibody production was determined by ELISA using OVA as an antigen. Antibody production was observed in the mice group immunized with F37A (plus MPL or CFA), but not in the group immunized with Peptide. (TIF) [file pone.0110425.s008.tif]

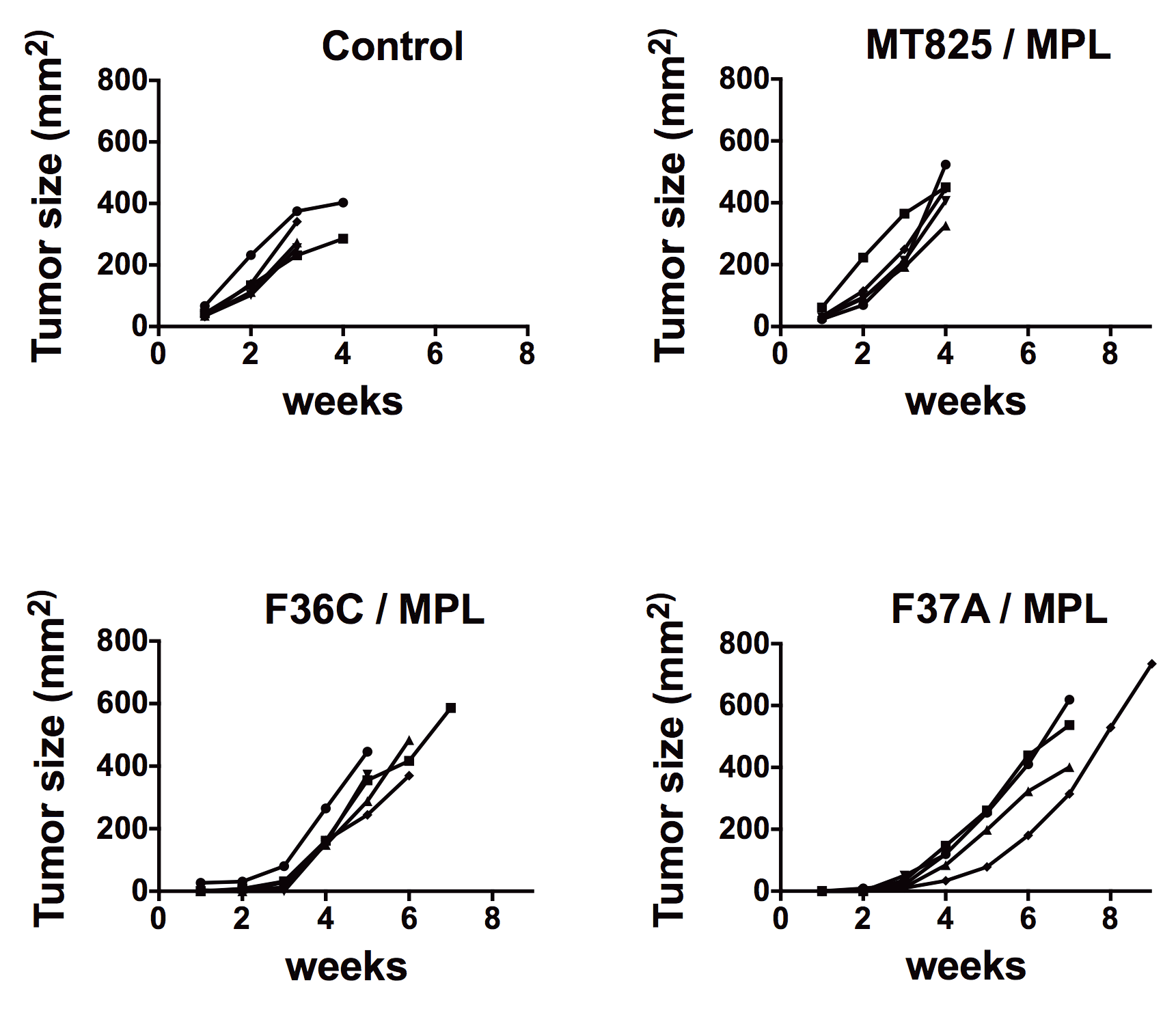

Supplement: Figure S9 — Tumor growth in mice immunized with F37A and F36C. Mice were intraperitoneally immunized with the indicated antigens plus MPL (n = 5 per condition). Following immunization, E.G7-OVA cells (2×10^6^ cells) were inoculated into the back of each mouse and growth of the tumor was monitored by measuring the tumor volume. Control mice were not immunized with any antigen. (TIF) [file pone.0110425.s009.tif]
